# Yo-Yo IR1-derived aerobic endurance machine-learning prediction in youth male soccer players using demographics information and resting heart rate variability parameters

**DOI:** 10.3389/fphys.2026.1904809

**Published:** 2026-09-11

**Authors:** Kacper Korzeniewski, Robert Makuch, Magdalena Mikielewicz, Maciej Rosoł, Jakub S. Gąsior, Marcel Młyńczak

**Affiliations:** 1 Faculty of Mechatronics, Institute of Metrology and Biomedical Engineering, Warsaw University of Technology, Warsaw, Poland; 2 Department of Physical Education, Faculty of Philology and Pedagogy, Casimir Pulaski University of Radom, Radom, Poland; 3 Department of Pediatric Cardiology and General Pediatrics, Medical University of Warsaw, Warsaw, Poland

**Keywords:** aerobic endurance, explainable AI, HRV, machine learning, Yo-Yo IR1

## Abstract

**Introduction/objective:**

This study evaluated the predictive value of demographic information and short-term resting heart rate variability (HRV) for estimating aerobic endurance fitness assessed via the Yo-Yo Intermittent Recovery Level 1 test in youth male soccer players.

**Methods:**

A total of 275 players (aged 8-18) were tested during pre-season. Data included players’ characteristics, resting heart rate and it’s variability in supine and standing positions, and Yo-Yo test results. Short-term (1–5 minutes) RR intervals (RRi) were recorded using the Polar Team 2, preprocessed for artifacts, and analyzed for time-, frequency-, and non-linear HRV features. Frequency-domain metrics were extracted from linearly detrended signals to ensure stationarity. Ten datasets combining player characteristics (age, stature, body mass, BMI, body composition, maturity offset, experience, match status) and HRV were used to train multiple machine learning (ML) regression models. Model performance was evaluated using standard metrics, and SHAP values identified key predictors.

**Results:**

The final sample included 237 players median (range) age and BMI were: 13 (8 – 18) years, 18.8 (13.8 – 29.3) kg/m^2^. The best models, Random Forest using 120-second supine data and Random Forrest using 240-second standing data respectively achieved RMSE 2.89 ml/kg/min (344 m), and MAE 2.27 ml/kg/min (270 m), and RMSE 2.82 ml/kg/min (336 m), and MAE 2.26 ml/kg/min (269 m). Compared to a demographics-only baseline for Random Forest returning RMSE of 3.15 ml/kg/min (375 m) and MAE 2.39 ml/kg/min (285 m), the inclusion of HRV features provided a modest but consistent improvement in predictive accuracy. Age, experience, maturity offset, and non-linear HRV features (symbolic dynamics, Higuchi’s Fractal Dimension) were most influential.

**Discussion:**

ML models combining basic characteristics and short-term HRV showed moderate performance. The incremental improvement attributable to HRV was modest, and these exploratory models are not currently ready to replace Yo-Yo field testing or guide individual training decisions.

## 1 Introduction

Aerobic endurance is recognized as one of the key determinants of soccer players’ performance (Helgerud et al., 2001). Numerous studies have demonstrated a positive correlation between maximal aerobic capacity and performance metrics, such as sprinting ability and total distance covered during match (Bradley et al., 2013; Karsten et al., 2016). Assessment of maximal oxygen uptake ( $\dot{V}O_{2\max}$) in soccer players: i) allows for the distinction between performance level as higher values have been observed in elite players compared to those at lower levels (Slimani et al., 2019), ii) is important to prescribe training programs individually to enhance performance optimally (Helgerud et al., 2001; Clemente et al., 2024), i.e., to optimize position-specific training loads and avoid overtraining (Alexiou and Coutts, 2008; Iaia et al., 2009), and iii) is valuable in talent identification, enabling scouts and coaches to recognize players with strong aerobic potential early on (Murr et al., 2018) Nevertheless, the gold-standard procedure to assess $\dot{V}O_{2\max}$ is expensive, time consuming, and cannot replicate the tactical, technical, and environmental demands that influence aerobic performance during match play. On the other hand, running-based field specific tests are considered unsuitable for regular use due to their interference with ongoing training schedules or matches (Schimpchen et al., 2023).

The endeavor would be to train models to predict/estimate aerobic endurance in young soccer players based on data that can be widely, rapidly and inexpensively collected at rest. Traditional methods have relied on direct laboratory measurements or simple field tests (Chamari et al., 2004, Chamari et al., 2005; Nobari et al., 2021). Some studies have explored the potential of combining multiple parameters such as biomarkers or anthropomorphic characteristics (Manou et al., 2018; Perroni et al., 2020). Although these studies demonstrated promising correlations to $\dot{V}O_{2\max}$, they remain preliminary and do not yet represent fully developed algorithmic prediction systems. This highlights a significant opportunity to develop and validate machine-learning-based (ML) models for accurate, non-invasive estimation of maximal oxygen uptake in youth soccer players. The ability to accurately estimate aerobic endurance without exhaustive endurance testing could greatly enhance how coaches design and individualize training programs, making them more responsive to each athlete’s physiological profile.

The main objective of this study was the internal development of a machine learning model for the preliminary prediction of aerobic endurance fitness measured by the Yo-Yo Intermittent Recovery Level 1 (IR1) test using demographic information alongside supine and standing resting HRV in youth male soccer players. From a technical point of view, the Yo-Yo-derived estimated $\dot{V}O_{2\max}$ was utilized as the model output; however, we do not assume that the model predicts actual physiological $\dot{V}O_{2\max}$. Additionally, the study aimed to evaluate the relative importance of the included demographic and HRV features to assess their incremental contribution to endurance modelling. Importantly, this research serves as an exploratory investigation in this cohort, rather than aiming to establish a replacement for individual field testing or a validated tool for individual training-load decisions.

## 2 Methods

This study was reported in accordance with the Transparent Reporting of a Multivariable Prediction Model for Individual Prognosis or Diagnosis (TRIPOD+AI) recommendations (Collins et al., 2024) and the Strengthening the Reporting of Observational Studies in Epidemiology (STROBE) guidelines (von Elm et al., 2007). Detailed checklists for both guidelines are provided in the **Supplementary Materials**.

### 2.1 Study population

A total of 275 male soccer players between 8 and 18 years old participated in the study. Inclusion criteria for study participation were: i) valid medical examination, ii) absence of musculoskeletal injury in the six months before the measurements, (ii) a minimum of one year of training experience, (iii) training at least 3 times a week. The study group consisted exclusively of male participants, as the data were collected from a soccer club that does not currently operate a girls’ section. Therefore, the absence of female players in the sample reflects the club’s structure rather than a deliberate exclusion. Participants were recruited for this study using a convenience sampling approach. Consequently, an *a priori* sample-size calculation was not performed prior to data collection; rather, the sample size was determined by the maximum number of participants available and eligible during the study period.

The study was conducted in accordance with the principles of the Declaration of Helsinki for research involving human subjects. Ethical approval was obtained from the appropriate ethics committee (approval number: KB/55/N02/2019). This 2019 clearance serves as an umbrella approval for the club’s ongoing routine physiological monitoring protocols, which encompassed the data collected in 2023 for this study. All participants and their legal guardians were fully informed about the purpose, procedures, potential benefits, and risks of the study. Written informed consent was obtained from the parents or legal guardians of the participants prior to the study.

### 2.2 Examinations

All examinations were conducted over a single week in 2023 during pre-season. Measurements took place exclusively during scheduled morning training sessions. The players and their parents (legal guardians) were instructed that players should maintain normal sleep behaviors (as usual during the 5 days before the examination), to refrain from vigorous physical activity and caffeine on the day before and the day of the study, to eat a normal, usual light breakfast, and to use the toilet (if necessary) on the day of the study before the examinations (Laborde et al., 2017). Anthropometric and body composition measurements and RR intervals (RRi) acquisition were carried out at least 1 h after breakfast at home. These examinations were performed in a quiet room with a stable, controlled temperature and humidity.

Demographic data, playing experience (years) and match participation status (starters or substitutes) were collected from the team coaches.

Anthropometric and body composition measurements were obtained using a Tanita MC-980 MA body composition analyzer. Participants were instructed to wear underwear and bare feet, maintain a standard upright standing posture with arms slightly separated from the torso to ensure proper bioelectrical impedance analysis. Prior the measurements, the device was calibrated according to the manufacturer’s guidelines to ensure measurement repeatability. Standing height (stature) was measured to the nearest 0.1 cm and body mass to the nearest 0.1 kg. Body mass index (BMI) was calculated as body mass divided by height squared (kg·m^−2^). Body composition variables included percentage and absolute fat mass (kg), fat free mass (kg), pure muscle mass, bone mineral mass, total body water, basal metabolic rate.

Moore’s equations were used to estimate maturity status (PHV) (Moore et al., 2015):


Maturity offset(years)=−7.999994+(0.0036124×age×stature).

However, we acknowledge the known limitations of maturity-offset equations, particularly their reduced accuracy in early- and late-maturing boys (Kozieł and Malina, 2018). This is an important consideration given the wide maturity-offset range of −4.2 to +3.6 years observed in our cohort.

RRi acquisition were conducted with participants wearing underwear. Firstly, in order to stabilize HR and respiratory rate, the players were asked to lie in the supine position for 5 minutes before the beginning of the appropriate registration. Secondly, RRi were registered over 10 minutes under controlled resting conditions: a) 5-minute, in a supine position, players remain still and refrain from speaking or moving; b) 5-minute with the players in a standing position. HRV parameters used for modeling were calculated (see 2.3. below) based on the data collected during both conditions.

Afterwards, players completed the Yo-Yo IR1 test (Deprez et al., 2014) supervised by a trained professional (RM), after which their Yo-Yo-derived estimated $\dot{V}O_{2\max}$ was calculated (Bangsbo et al., 2008) using following equitation:


$$YoYo\;IR1\;derived\;\dot{V}O2\text{max}\left(\text{mL}*{\text{kg}}^{-1}*min^{-1}\right)=IR1\;distance\left(m\right)\times 0.0084+36.4$$

This stage provided the aerobic endurance fitness data necessary for model development and validation.

### 2.3 RR intervals acquisition, processing and analysis of heart rate variability

RRi were collected using HR monitor and the Polar Team 2 system, which enables real-time transmission of physiological data via Bluetooth. Polar H10 HR monitor (chest-strap, Polar Electro Oy) was moisturized to provide proper signal conduction and placed tightly and comfortably below the chest muscles whilst seated, then athletes were asked to lay supine before HR and respiratory rate stabilization period (Giles et al., 2016, GilesDraper, 2018). The RRi were exported as.txt (ASCII format) files. Then, RRi were imported into a CoRRection software program designed for HRV data preprocessing (Mikielewicz et al., 2025) to review, identify and correct technical artifacts. Technical artifacts were identified as one of seven types of errors according to the identification procedure recommendations (Giles et al., 2016, GilesDraper, 2018). Correction was performed for T2-T5 and T6b artifacts, T1 and T6a artifacts were not corrected since it is not possible to identify both artifacts without simultaneous ECG recordings (Cilhoroz et al., 2020). Technical artifacts were corrected: one RRi before, and one after each technical artifact were eliminated and replaced by RRi computed by interpolation of degree zero based on the surrounding RRi (Peltola, 2012). Researchers (MM and JSG) responsible for the RRi visual inspection and artifact correction were blinded to the players’ characteristics. Data of players with RRi which required correction of over 5% of acquired time series were excluded from statistical analysis.

Screened and corrected RRi time series were segmented into multiple time windows. Specifically, each 5-minute RRi was divided into sequential segments of 1, 2, 3, 4, and 5 minutes. Precisely, the first segment included only the first minute, the second included the first two minutes, the third included the first three minutes of signal and so on, up to the full 5-minute duration. This approach allowed to assess how the duration of the analyzed signal influences the quality of predictions.

The Python library Neurokit2 (Makowski et al., 2021) in version 0.2.13 was utilized to calculate HRV parameters (researchers KK and MR; see parameters description and details in **Supplementary Table 1**).

Subsequently, input datasets for ML models were prepared. For each RRi time series length (1–5 minutes) and both conditions (supine and standing position) the time domain, and non-linear HRV parameters were calculated (Buccelletti et al., 2012). Before calculating frequency-domain HRV indices, linear detrending was performed in order to achieve stationarity (Wu et al., 2007). Stationarity was assessed using the Kwiatkowski–Phillips–Schmidt–Shin (KPSS) tests (Kwiatkowski et al., 1992), with the significant level set to 0.05. To obtain a continuous signal for spectral analysis, the discrete sequence of RRi was interpolated at a resampling rate of 100 Hz. Power spectral density (PSD) was estimated using Welch’s method. Prior to PSD estimation, the interpolated signal underwent constant detrending (zero-mean subtraction). We applied a Hann window with a 50% overlap, with the window length automatically determined to capture at least two cycles of the lowest frequency band of interest. Following the default NeuroKit2 pipeline, the output power spectra were normalized, yielding relative, unitless power values rather than absolute power. The frequency domain HRV parameters were computed from the detrended signals and incorporated into the feature dataset. During the calculation of HRV parameters, if any feature resulted in infinite (inf) values for any participant, the entire column corresponding to that feature was deleted from the dataset to ensure mathematical stability (as detailed in **Supplementary Table 2**).

This process resulted in the creation of 10 distinct datasets. Additionally, dataset containing only demographic and anthropometric variables (age, stature, body mass, BMI, maturity offset, body composition, experience and match status (starter); see **Supplementary Table 1** for the full list of parameters and **Supplementary Table 2** for the details of each dataset) was created. There were no missing values across the demographic, anthropometric, or body composition data; therefore, no data imputation procedures were required to handle missingness. Results obtained for this dataset served as a baseline, enabling assessment of the incremental predictive value of HRV-derived features beyond demographic and anthropometric variables.

### 2.4 Machine learning algorithm and features importance

Based on the collected dataset, we aimed to evaluate the accuracy of endurance predictions using players’ demographic data and HRV parameters derived from RRi recorded under resting conditions.

The datasets were divided into training and testing subsets using 75:25 ratio with age-stratified sampling to ensure a balanced distribution of participants across age group in both subsets. Importantly, the specific assignment of participants to the training and testing sets was kept identical across all datasets. Feature standardization was performed using statistics (mean and standard deviation) calculated from the training set only, to prevent data leakage and ensure the integrity of the model evaluation process.

Different ML algorithms, commonly used for regression problems, were utilized: Linear Regression, Support Vector Machine, Decision Tree, Random Forest and Gradient Boosting for Regression (Breiman, 2001; Noble, 2006; Su et al., 2012; Natekin and Knoll, 2013). The hyperparameter tuning was performed for each algorithm using the grid-search technique combined with the 10-fold cross validation on training set. In each iteration of the validation, metrics like mean absolute error (MAE), R2 score, root mean squared error (RMSE) were calculated (Botchkarev, 2018). Following hyperparameter tuning, the optimal parameter set was selected based on the lowest RMSE. Final models were then trained on the entire training sets using the selected hyperparameters and subsequently evaluated on the independent test sets, which were not used during either model training or hyperparameter optimization. The best model for each dataset was determined based on the lowest RMSE score.

The comparison of recording windows and final model configurations was performed on the held-out test sets and should therefore be interpreted as exploratory rather than confirmatory. No nested cross-validation or separate validation set was used for recording-window selection.

Additionally, the performance of models trained on a baseline dataset containing only demographic, anthropometric, body composition, and maturity-related variables was compared with models incorporating HRV features to evaluate the incremental predictive value of HRV. The contribution of HRV was quantified as the change in model performance between baseline and results obtained for HRV-enhanced models.

To assess the importance of individual features used in the machine learning models, explainable artificial intelligence (XAI) techniques were applied. Specifically, the SHAP Python package (Lundberg and Lee, 2017) was used to perform model-level variable importance analysis. This involved calculating SHAP values on the test set, which quantify the impact of each feature on model output. The results were visualized using importance plots to facilitate interpretation.

**Figure 1:**
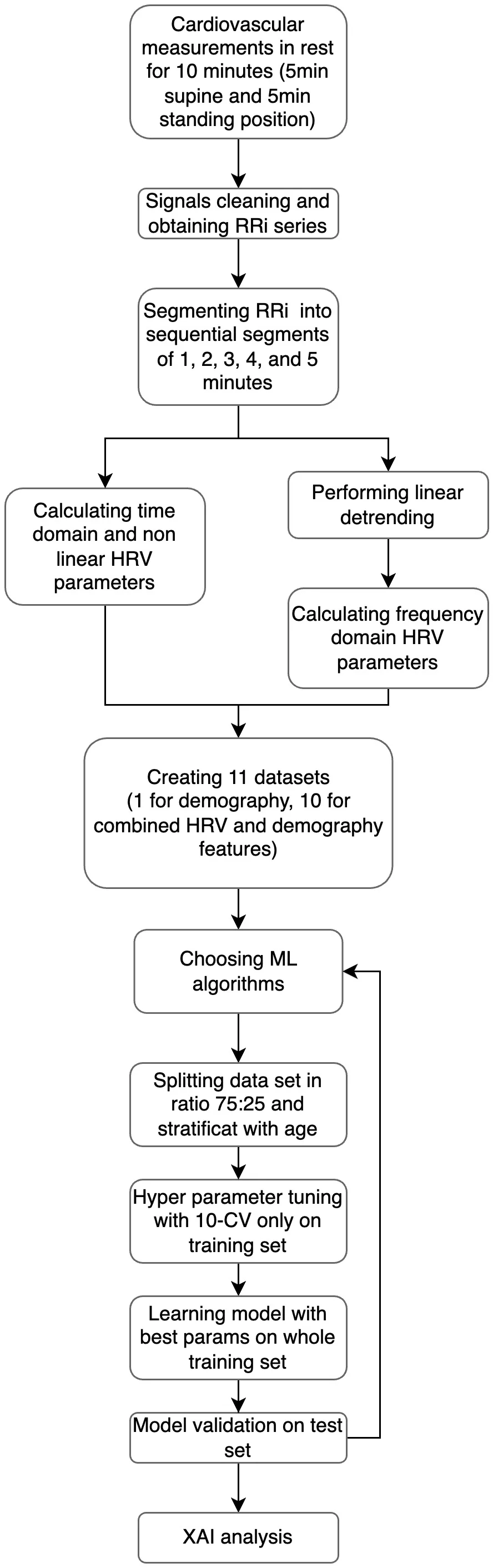
Diagram presenting the individual steps of the conducted analysis.

For all analysis Python 3.11.11 was used. A full diagram of the performed analysis is presented in **Figure 1**.

## 3 Results

Recordings requiring correction of more than 5% of RRi were excluded, resulting in the removal data of 38 players (13.8%).

The final sample included 237 male youth soccer players – 128 (54%) starters and 109 (46%) substitutes. The players’ median (range) age, stature, body mass, BMI, basal metabolic rate, body fat percentage, body fat mass, fat-free mass, total body water, normalize bone mass, pure muscle mass, maturity offset and playing experience were as follows: 13 (8 – 18) years, 163.3 (128.1 – 190.4) cm, 49.6 (23.9 – 85.8) kg, 18.8 (13.8 – 29.3) kg/m^2^, 6418.4 (4668.3 – 9692.5) kJ/day, 16.3 (7.6 – 37) %, 7.9 (3.3 – 23.8) kg, 39.7 (19.7 – 72.9) kg, 29.4 (14.4 – 50.1) kg, 2.1 (1.1 – 3.6) kg, 37.6 (18.6 – 69.3) kg, -0.1 (-4.2 – 3.6) years and 3 (1 – 9) years. Yo-Yo IR1 performance was: 920 (280 – 3080) m, i.e. median Yo-Yo IR1-estimated $\dot{V}O_{2\max}$ of 44.1 (38.8 – 62.3) ml/kg/min.

To assess potential selection bias due to the exclusion of 38 players, a formal comparison with the 237 retained players was conducted (using the Mann-Whitney U test for continuous variables). The excluded group exhibited a median (IQR) age of 12.0 (11.0–13.8) years, stature of 168.4 (152.7–175.8) cm, body mass of 51.2 (37.8–62.3) kg, BMI of 17.8 (16.0–20.1) kg/m^2^, maturity offset of 0.2 (-1.9–1.2) years, and Yo-Yo IR1 distance of 720.0 (520.0–1160.0) m.

In comparison, the retained group demonstrated medians (IQR) of 13.0 (11.0–14.0) years for age (p = 0.271), 163.3 (150.4–175.7) cm for stature (p = 0.725), 49.6 (39.6–63.6) kg for body mass (p = 0.527), 18.8 (17.1–21.3) kg/m^2^ for BMI (p = 0.056), -0.1 (-1.6–1.3) years for maturity offset (p = 0.644), and 920.0 (600.0–1440.0) m for Yo-Yo IR1 distance (p = 0.174). While minor differences were noted, exclusions were strictly triggered by the objective RR-interval artifact threshold (>5%). However, residual selection bias cannot be completely excluded.

The unified dataset of 237 participants was split into a training set (n=177) and a held-out test set (n=60). To guarantee strict reproducibility of the dataset partitions and model initializations, a global random seed of 67 was fixed across all code implementations.

The percentage of stationary data, based on the results of the KPSS test conducted on RRi signals before and after linear detrending, is presented illustratively in **Table 1**. The results indicate that linear detrending effectively addressed the issue of non-stationarity in the data and thereby enabling reliable calculation of frequency-domain HRV features. Additionally, a trend was noted with respect to body position: signals recorded in the standing position exhibited a higher proportion of stationarity compared to those recorded in the supine position.

**Table 1:** Stationarity data based on KPSS test.

Time	Supine		Standing	
	Raw	Detrend	Raw	Detrend
60s	39.24%	98.73%	40.51%	100.00%
120s	49.37%	99.16%	49.79%	99.58%
180s	48.10%	95.36%	67.51%	100.00%
240s	53.16%	96.20%	64.98%	99.58%
300s	47.26%	95.78%	65.40%	100.00%

The performance metrics obtained for the best performing algorithm in terms of the lowest RMSE and R2 for each dataset were shown in **Table 2**, along with the corresponding model names and dataset identifiers (refers to the specific measurement condition, defined by the athlete’s body position and the length of the RR interval recording window).

**Table 2:** Lowest RMSE (in ml/kg/min) and R2 values achieved for each dataset and ML model type.

Supine position					
	60s	120s	180s	240s	300s
Linear regression	5.40 (-0.21)	3.54 (0.48)	3.81 (0.40)	3.82 (0.39)	3.89 (0.37)
SVM	3.46 (0.50)	3.40 (0.52)	3.24 (0.56)	3.27 (0.55)	3.49 (0.49)
Decision Tree	3.71 (0.43)	3.42 (0.51)	3.38 (0.52)	3.14 (0.59)	3.61 (0.46)
Random Forest	2.94 (0.64)	2.89 (0.65)	3.00 (0.63)	2.97 (0.63)	3.00 (0.63)
Gradient Boosting	3.60 (0.46)	3.17 (0.58)	3.66 (0.44)	3.16 (0.58)	3.84 (0.39)
Standing position					
	60s	120s	180s	240s	300s
Linear regression	3.80 (0.40)	3.71 (0.43)	3.89 (0.37)	4.85 (0.02)	4.80 (0.04)
SVM	3.85 (0.38)	3.43 (0.51)	3.38 (0.52)	4.12 (0.29)	4.25 (0.25)
Decision Tree	4.17 (0.28)	3.04 (0.61)	3.39 (0.52)	3.48 (0.50)	3.34 (0.54)
Random Forest	3.03 (0.62)	2.86 (0.66)	2.87 (0.66)	2.82 (0.67)	2.89 (0.65)
Gradient Boosting	3.62 (0.46)	3.06 (0.61)	3.29 (0.55)	3.21 (0.57)	3.32 (0.54)
Baseline model (demography and maturity data)					
Linear regression	3.09 (0.60)				
SVM	3.29 (0.55)				
Decision Tree	3.17 (0.58)				
Random Forest	3.15 (0.59)				
Gradient Boosting	3.45 (0.50)				

**Figure 2:**
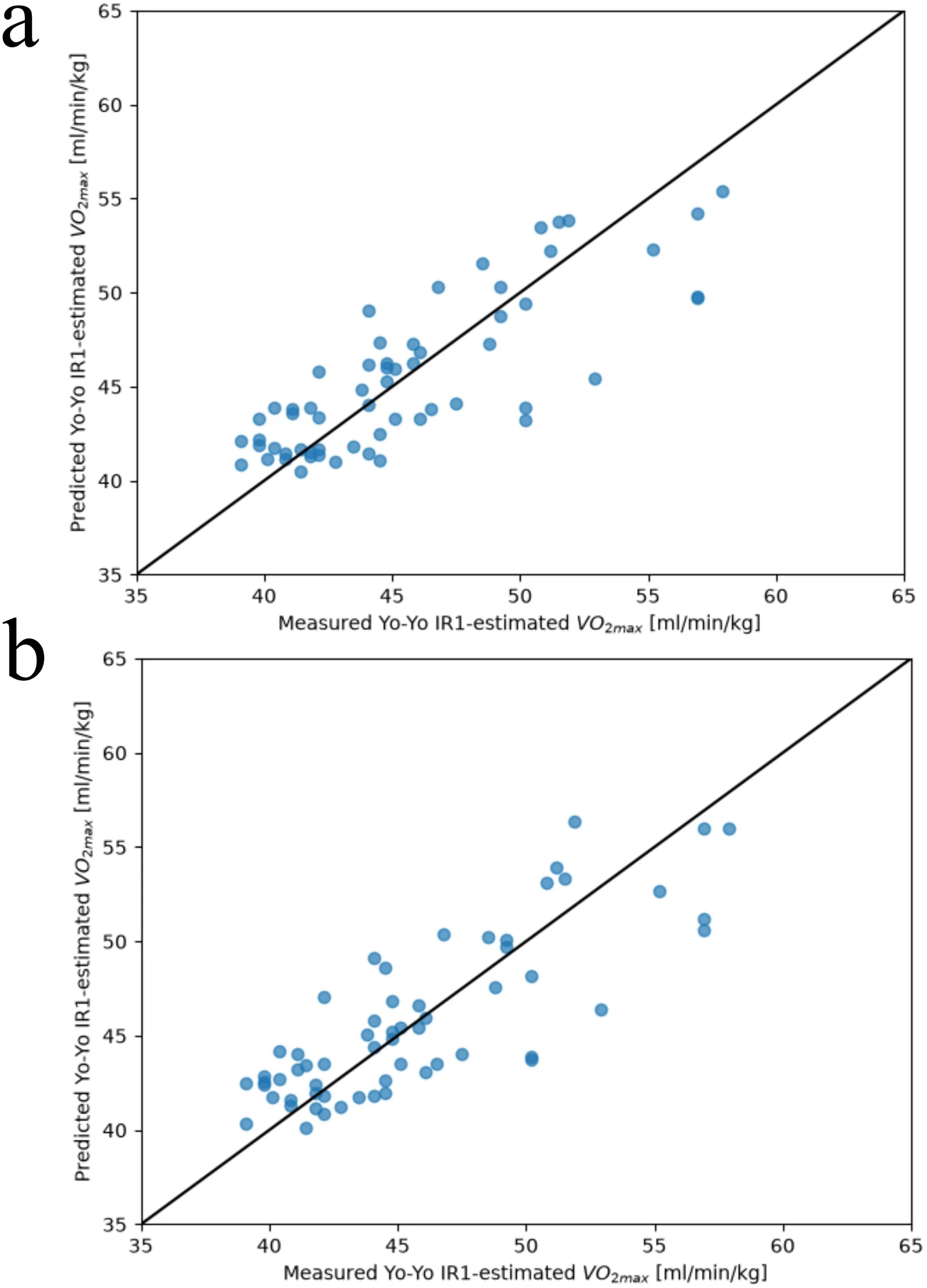
The plot of Yo-Yo IR1-estimated and predicted $\dot{V}O_{2\max}$ values. The solid black line represents the value where predicted value is equal to the measured one. **(A)** Prediction of Random Forest model trained on dataset calculated from 120s supine data. **(B)** Prediction of Random Forest model trained on dataset calculated from 240s standing data.

**Figure 3:**
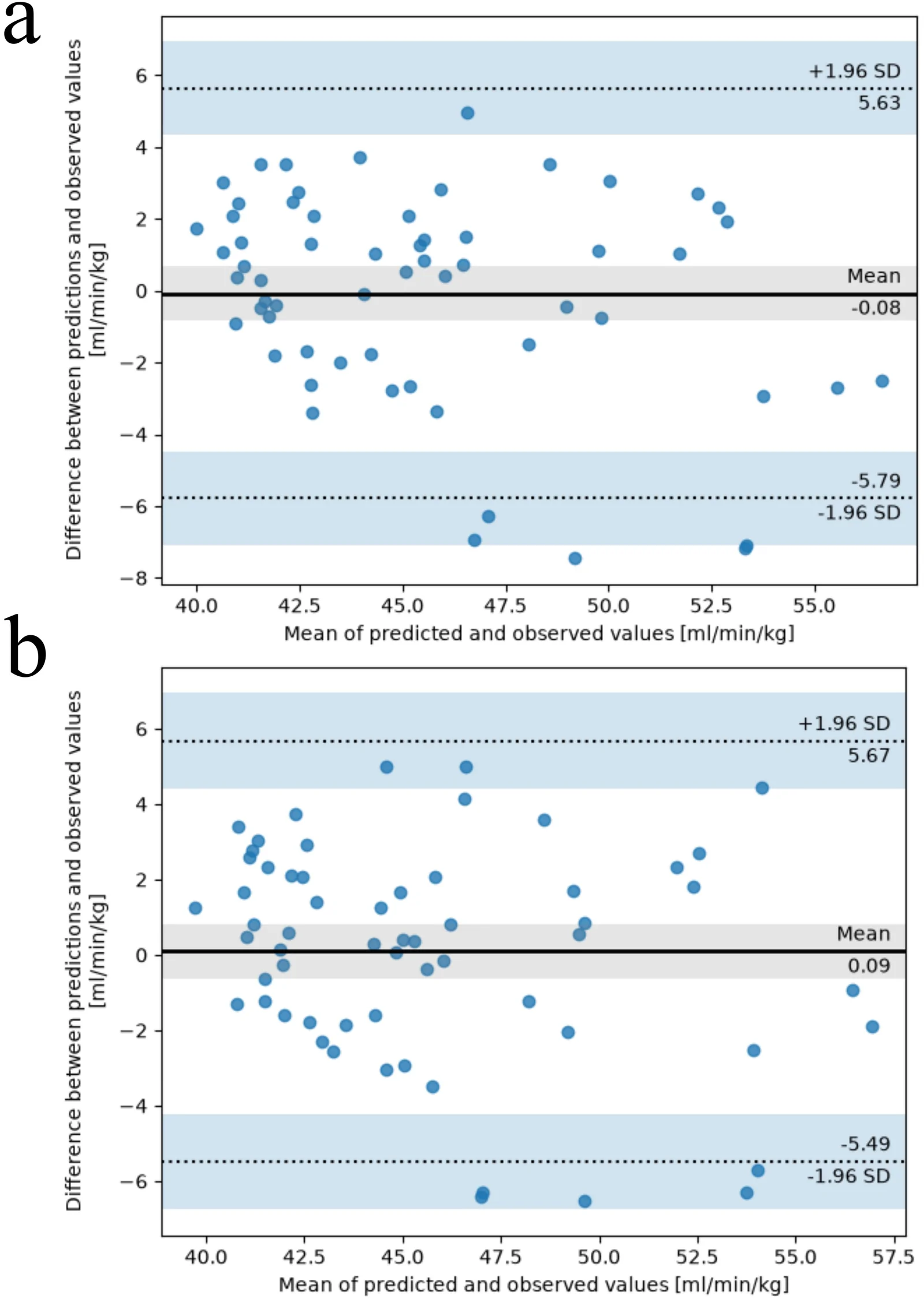
The Bland-Altman plot of Yo-Yo IR1-estimated predicted $\dot{V}O_{2\max}$ value based on the **(A)** Result of analyses for Random Forest model trained on dataset calculated from 120s supine data. **(B)** Result of analyses for Random Forest model trained on dataset calculated from 240s standing data.

**Figure 4:**
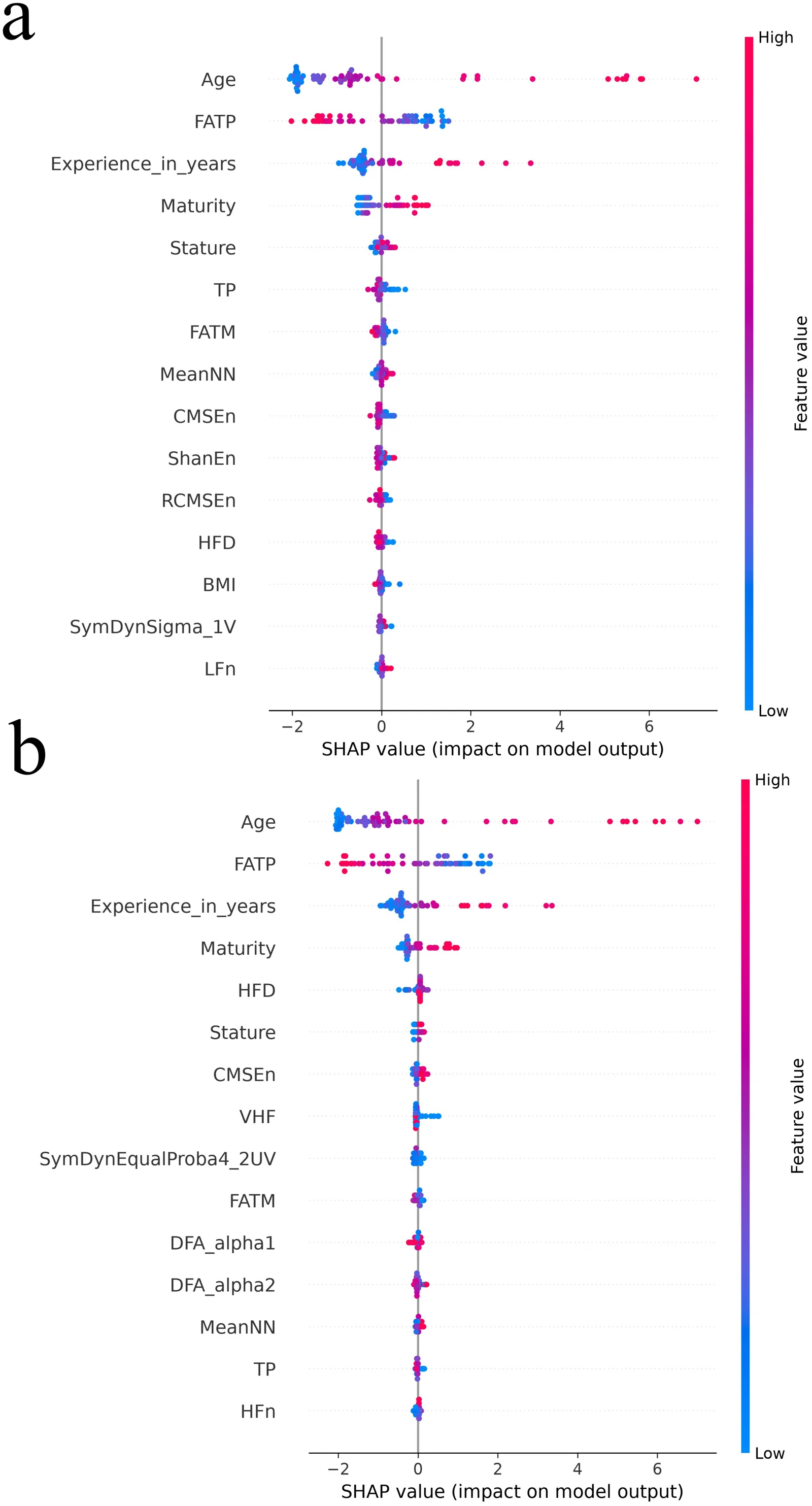
Feature importance (Shapley values) for the best model in each dataset group. **(A)** Result of XAI analyses for Random Forest model trained on dataset calculated from 120s supine data. **(B)** Result of XAI analyses for Random Forest model trained on dataset calculated from 240s standing data.

The best performing models for each group were as follows: a Random forest model trained on 240 second standing data and the Random Forest model trained on 120 second supine data. For these two models the Yo-Yo IR1-estimated $\dot{V}O_{2\max}$ values and the corresponding predictions are presented in **Figure 2**. The Bland-Altman plots for these models are presented in **Figure 3**. Additionally, Shapley values (SHAP) representing feature importance are visualized in **Figure 4**.

We observed that, similar key features were important for model performance in both cases. Demographic variables such as Age, Experience in years, Body Fat Percentage (FATP) and Maturity consistently played a crucial role in prediction accuracy. Additionally, among the HRV parameters, features from the symbolic dynamics group and entropies group from the nonlinear domain emerged as significant contributors in both conditions.

Furthermore, the visualization presented in **Figure 4** provides qualitative insights into how individual variables influence the model’s predictions. For instance, a higher maturity offset-indicating that an individual is further past their peak height velocity is associated with an increased predicted aerobic endurance value. In contrast, higher spectral power in the very high frequency (VHF) band is linked to lower aerobic endurance predictions. This suggests that while biological maturity positively contributes to aerobic capacity estimation, elevated VHF power may reflect physiological patterns that negatively impact model response.

For datasets built based on data measured in supine position, the lowest RMSE equal to 2.89 ml/kg/min (344 m) was observed for recording of 120 seconds. This model reaches also MAE equal to 2.27 ml/kg/min (270 m) and R2 equal to 0.65 on the test dataset. For datasets built based on data measured in standing position The lowest RMSE equal to 2.82 ml/kg/min (336 m) was observed for recording of 240 seconds taken in the standing position. This model reaches also MAE equal to 2.26 ml/kg/min (269 m) and R2 equal to 0.67 on the test dataset. However, the differences between adjacent recording windows were small. Therefore, these configurations should be interpreted as the best-performing configurations within the present exploratory comparison, rather than as definitively superior recording durations.

To establish baseline performance, the best overall demographic-only model was Linear Regression achieving an RMSE of 3.09 ml/kg/min (368 m) and an R2 of 0.60. This model reaches also MAE equal to 2.45 ml/kg/min (292 m). While a demographic-only Random Forest achieved an RMSE of 3.15 ml/kg/min (375 m), R2 of 0.59 and MAE 2.39 ml/kg/min (285 m). This model was used as the same-learner comparator to explicitly estimate the incremental contribution of HRV.

The incorporation of HRV-derived features from data collected in the supine position reduced the RMSE by 0.26 ml/kg/min (31 m) and increased the R2 score by 0.06 while features derived from standing position reduce the RMSE by 0.33 ml/kg/min (39 m) and increased the R2 score by 0.08. In this analysis, a Random Forest model was used for the demographic-only baseline to reduce the degrees of freedom and ensure consistency with the model type chosen for the combined demographic and HRV datasets.

To further evaluate the prediction accuracy and agreement of the best-performing models, a Bland-Altman analysis was conducted on the test set.

For the Random Forest model using the Supine 120s dataset, the mean bias was -0.080 ml/kg/min (95% CI: -0.833 to 0.672), indicating the overall mean difference between the model’s predictions and the Yo-Yo IR1-derived estimated $\dot{V}O_{2\max}$. The upper limit of agreement was 5.626 ml/kg/min (95% CI: 4.324 to 6.929), and the lower limit of agreement was -5.787 ml/kg/min (95% CI: -7.090 to -4.485).

Similarly, for the Random Forest model using the Standing 240s dataset, the mean bias was 0.092 ml/kg/min (95% CI: -0.643 to 0.827). The upper limit of agreement was 5.671 ml/kg/min (95% CI: 4.397 to 6.944), and the lower limit of agreement was -5.486 ml/kg/min (95% CI: -6.760 to -4.213).

Although visual inspection of the plots might suggest a negative trend, a simple linear regression analysis between the mean of the two methods and their differences was performed to formally test for proportional bias. This analysis revealed a significant bias for the Supine 120s model (p = 0.0183, slope = -0.2056) but no significant bias for the Standing 240s model (p = 0.0664, slope = -0.1541). The negative slope observed for the Supine 120s model indicates a tendency to overestimate lower values and underestimate higher values of aerobic capacity, demonstrating that prediction error varies systematically across the range of observed $\dot{V}O_{2\max}$ values For the Standing 240s model, prediction errors remained relatively consistent across different levels of actual aerobic performance.

To verify the robustness of our findings, a sensitivity analysis was conducted on the best-performing model for the supine and standing datasets. First, to investigate the potential influence of baseline HR on the reliability of the VHF band, we examined the relationship between VHF power and MeanRR (which is inversely related to resting HR). A correlation analysis was performed to determine whether slower HR and thus a lower sampling density of R-peaks compromised the VHF estimation. For the dataset 120 second supine, we observed no significant correlation between VHF power and MeanRR (Pearson r = -0.089, p = 0.174; Spearman rho = -0.086, p = 0.186). Similarly, for the 240-second standing dataset, no significant correlation was found (Pearson r = 0.030, p = 0.644; Spearman rho = -0.013, p = 0.842). These results indicate that baseline HR did not systematically bias the VHF measurements in our sample under either recording condition.

Despite the lack of correlation, we re-evaluated the model after excluding the Very High Frequency (VHF) band from the feature set to assess whether it was actively contributing to predictive accuracy. For 120 second supine dataset, the model without VHF yielded an RMSE of 2.88 ml/kg/min (343 m), an MAE of 2.21 ml/kg/min (263 m), and an R2 of 0.65 on the test set. The model for 240 second standing dataset, without VHF yielded an RMSE of 2.81 ml/kg/min (335 m), an MAE of 2.27 ml/kg/min (270 m), and an R2 of 0.67 on the test set. As these results show, excluding the VHF band did not degrade model performance. These results may confirm that standard frequency bands are sufficient for this analysis, and that the VHF band does not provide meaningful predictive value and may even introduce unnecessary noise in ultra-short 120-second recordings and short 240 recordings.

## 4 Discussion

The application of ML models to predict aerobic endurance fitness, estimated by the Yo-Yo IR1 test, in youth male soccer players using demographic and anthropometric characteristics but also HRV parameters recorded at rest, was investigated. Within this single-center internal development study, we demonstrate that models combining demographic data with short-term resting HRV achieved moderate predictive performance for Yo-Yo-derived aerobic fitness.

Earlier studies have investigated aerobic endurance estimation using wearable devices as an alternative to traditional laboratory or field-based testing. Düking et al (Düking et al., 2024). evaluated smartwatch-derived estimates against actual $\dot{V}O_{2\max}$ measured directly via respiratory gas analysis. They concluded that the agreement between the wearable devices and the laboratory reference was limited, highlighting the difficulty of precise individual estimation. Furthermore, these wearable methods rely on hidden, proprietary algorithms and require extended periods of physical activity, such as at least 10 minutes of continuous running. This makes them less convenient and potentially impractical for routine use compared to resting measurements, especially in settings where time, equipment, or physical readiness may be limited.

The baseline comparison revealed that the addition of short-term resting HRV features provided limited incremental predictive value beyond demographic, anthropometric, and maturity-related variables. However, this finding raises an important question regarding the extent of shared physiological information captured by these predictors. Although several non-linear HRV features, particularly from the symbolic dynamics domain, were among the most influential variables according to SHAP analysis, the overall improvement in predictive performance remained modest. One possible explanation is that, in youth male soccer players aged 8–18 years, maturation of the autonomic nervous system may be closely associated with somatic growth and biological maturation, resulting in overlapping predictive information. Consequently, the value of the proposed ML approach may extend beyond prediction alone by providing insights into the relative contribution of different physiological and developmental factors associated with aerobic endurance performance.

Despite the modest overall improvement in global error metrics, specific non-linear HRV parameters were consistently identified by the SHAP analysis as highly influential contributors to the model’s decisions. These findings align with established physiological principles, particularly the observation that well trained individuals typically exhibit lower resting heart rate, i.e., making time domain HRV parameters logical and informative predictors (D’Souza et al., 2014). Moreover, our results highlight the relevance of nonlinear HRV parameters, which are increasingly recognized for their ability to capture the complexity of autonomic regulation and cardiovascular adaptability in young athletes (Voss et al., 2009). This nonlinear approach offers distinct advantages over traditional linear HRV metrics, particularly under non-stationary conditions such as exercise or recovery, where standard methods may lose validity.

Symbolic dynamics facilitates the classification of short RR interval sequences into specific patterns that reflect autonomic balance. These insights are of relevance in sports contexts, where rapid shifts in autonomic tone occur and where understanding these shifts can support individualized training load adjustments, fatigue monitoring, and performance optimization. Furthermore, the independence of symbolic dynamics from respiratory rate changes serves to ensure its reliability, even in circumstances where respiratory data is either unavailable or subject to variation (Gąsior et al., 2024). In addition, currently entropies are rarely used in athlete assessment, despite its potential to reveal subtle autonomic irregularities. More research is needed to explore its value in sports science and its possible role in monitoring training, fatigue, and recovery.

The predicative value of HRV features supports previous research indicating a moderate correlation between HRV and aerobic capacity (Pereira et al., 2019). Additionally, demographic variables such as age, body mass index, body fat percentage and maturity offset emerged as importance predictors of endurance performance. This is consistent with the understanding that these factors evolve with growth and training progression, influencing physiological development and endurance potential (Mota et al., 2002; Enríquez-del-Castillo et al., 2022).

Traditionally, a 5-minute recording has been considered as the gold standard for reliable short term HRV analysis (Kleiger et al., 2005). However, our findings suggest that shorter recordings can also be useful for predictive modeling. Specifically, 2-min and 4-min short-term HRV provided the one of the best performances in our ML modeling. This suggests that valuable information can be derived from shorter recordings.

Interestingly, the ML models provided qualitative insights, such as the observation that higher spectral power in the Very High Frequency (VHF) band is a negative predictor of Yo-Yo derived aerobic endurance fitness. The VHF band (>0.4 Hz) is largely influenced by rapid respiratory frequencies (Chang et al., 2014). Saboul et al (Saboul et al., 2014). demonstrated that many athletes breathe spontaneously below 0.15 Hz, while other studies presented relation of frequency domain HRV to resting heart rate (Ziadia et al., 2023). Furthermore, from a signal-processing perspective, elevated VHF may also capture micro-movements or restlessness during the resting measurement. In both contexts, the ML algorithm may have correctly recognized this high-frequency noise or rapid respiratory pattern as a biomarker of lower aerobic capacity. However, since respiration and movement were not directly recorded in this study, the interpretation of VHF as a marker of tachypnea or restlessness remains an explicit *post hoc* hypothesis. Future studies incorporating direct respiratory and kinematic monitoring are required to confirm this mechanistic link.

Importantly, methodological approach calculating time domain and nonlinear HRV parameters from no detrended RRi signals, while deriving frequency domain features from detrended signals was implemented as a principled *a priori* choice. Since linear detrending alters the temporal structure and statistical properties of the raw RRi series (what appears to be a necessary mathematical transformation to achieve stationarity for valid frequency-domain analysis), it is crucial to apply this step selectively. This reinforces the need for a transparent preprocessing strategy, where frequency-domain features are derived from detrended signals, while time-domain and non-linear parameters are calculated from raw data to preserve their true physiological meaning.

Despite the promising results, this study has several limitations that should be considered. The relatively small number of participants may limit how well the machine learning models apply to other groups. A larger dataset would likely improve the accuracy and reliability of the results. Although we used well-known machine learning methods, trying more advanced or combined models could reveal deeper patterns and improve predictions. All participants were male soccer players from one training center. This lack of diversity may affect how well the findings apply to other athletes. Including players from different clubs and regions would make the results more broadly useful. It is important to examine whether the same patterns hold true for female players to create more inclusive models. Direct measured $\dot{V}O_{2\max}$ values were not used in this study. The estimated aerobic endurance fitness was derived from the Yo-Yo test as the reference for model training and evaluation. While the Yo-Yo test is a practical and widely used field method, it may not provide the same level of accuracy as direct $\dot{V}O_{2\max}$ obtained through laboratory-based cardiopulmonary exercise testing (Martínez-Lagunas and Hartmann, 2014). Consequently, the presence of estimation error in the reference values may affect the precision of the model predictions. Incorporating directly measured $\dot{V}O_{2\max}$ values in future studies could enhance the robustness and validity of the predictive models. Moreover, this study is marred by an absence of data regarding respiration rate. The incorporation of features pertaining to respiration rate variability has the potential to enhance the accuracy of Yo-Yo IR1-estimated $\dot{V}O_{2\max}$ predictions. Devices such as the Pneumonitor 3, which are capable of recording both ECG and respiratory signals, offer a promising solution for future research in this field (Młyńczak et al., 2017).

Moreover, the presence of statistically significant proportional bias in the Supine 120s model may reflect a regression-to-the-mean effect, which is commonly observed in ensemble tree-based machine learning models. As a result, predictions at the extremes of the $\dot{V}O_{2\max}$ distribution should be interpreted with caution. Furthermore, while the models demonstrated near-zero mean bias, the limits of agreement span approximately 11.4 ml/kg/min (roughly 1360 m of Yo-Yo IR1 distance). This wide individual variability indicates that while the models perform well at a group level, individual predictions should be interpreted with caution.

An additional limitation concerns model and recording-window selection. Although hyperparameter tuning was performed within the training set only, the comparison of algorithms and recording windows was based on held-out test-set performance. Therefore, the highlighted configurations should be interpreted as exploratory and may be affected by selection optimism. In particular, the small differences in RMSE, MAE, and R^2^ between adjacent recording windows do not establish a reliable ranking of recording durations. Future studies should use nested or repeated cross-validation, a separate validation set for configuration selection, or external validation to confirm the optimal model and recording duration.

A notable limitation of this study is the high predictor-to-sample ratio. Specifically, the analysis utilized approximately 80 predictors relative to 177 training observations. This high dimensionality compared to the sample size introduces a risk of overfitting, as models may begin to capture noise rather than underlying physiological patterns. Although this risk was mitigated by utilizing tree-based machine learning algorithms which are inherently more robust to high-dimensional datasets and multicollinearity, the potential for overfitting cannot be entirely eliminated. Future research should aim to validate these findings using larger cohorts to achieve a more favorable predictor-to-observation ratio.

Another limitation of this study is the estimation of biological maturity. As sitting height was not measured, maturity offset was calculated using the age-by-stature equation proposed by Moore et al. (2015). Although this is a validated and practical approach, the absence of sitting height may reduce the accuracy of maturity estimation (Kozieł and Malina, 2018), and some estimation error cannot be excluded.

Furthermore, we did not quantify sampling uncertainty for our performance metrics using methods such as paired bootstrapping or repeated-resampling confidence intervals. Because model configurations and recording windows were compared directly on the held-out test set, introducing potential selection optimism, the lack of confidence intervals further underscores that our reported point estimates must be interpreted strictly as exploratory.

Lastly, the age range of the participants was not evenly spread. A better balance of age groups would help us understand more clearly how age and development affect the relationship between heart rate signals, personal data, and endurance performance.

By extending the number of participants and sites from which the data was gathered, the next model may be more generalized.

## 5 Conclusion

Within this single-centre cohort, internally developed models combining demographic and anthropometric variables with short-term resting HRV yielded test-set RMSEs of 2.82-2.89 mL·kg^−1^·min^−1^ and MAE of 2.26-2.27 mL·kg^−1^·min^−1^ explicitly measured on test set. Compared with the same-learner demographic-only Random Forest baseline, the observed improvements reduced the RMSE by 0.26 and 0.33 mL·kg^−1^·min^−1^ when incorporating supine and standing HRV features, respectively. Because algorithms and recording windows were compared on the held-out test set, performance uncertainty was not quantified, individual limits of agreement were wide, spanning approximately 11.4 mL·kg^−1^·min^−1^ providing context for the substantial individual-level error despite the lower MAE, and proportional bias was present in the Supine 120-s model, these findings should be considered exploratory. External validation and prespecified model-selection procedures are required before individual or applied use.

## Data Availability

The datasets presented in this article are not readily available because they contain personal or sensitive information about participants but are available from the corresponding author on reasonable request. Requests to access the datasets should be directed to jakub.gasior@wum.edu.pl.
