# Supplementary material for: Yo-Yo IR1-derived aerobic endurance machine-learning prediction in youth male soccer players using demographics information and resting heart rate variability parameters
Source: Front Physiol. 2026 Sep 11;17:1904809. doi: 10.3389/fphys.2026.1904809 (PMC13613047; doi:10.3389/fphys.2026.1904809)
Supplement: Supplementary file 1 [file SupplementaryFile1.docx]

Supplementary Material

# 1 Adherence to Reporting Guidelines

STROBE (Strengthening the Reporting of Observational Studies in Epidemiology)

|  | Item | Recommendation | Section |
| --- | --- | --- | --- |
| **Title and abstract** | 1 | (*a*) Indicate the study’s design with a commonly used term in the title or the abstract | Title and Abstract |
|  |  | (*b*) Provide in the abstract an informative and balanced summary of what was done and what was found | Abstract |
| Introduction |  |  |  |
| Background /rationale | 2 | Explain the scientific background and rationale for the investigation being reported | Introduction |
| Objectives | 3 | State specific objectives, including any prespecified hypotheses | Introduction |
| Methods |  |  |  |
| Study design | 4 | Present key elements of study design early in the paper | Study Population |
| Setting | 5 | Describe the setting, locations, and relevant dates, including periods of recruitment, exposure, follow-up, and data collection | Examinations |
| Participants | 6 | (*a*) *Cohort study*—Give the eligibility criteria, and the sources and methods of selection of participants. Describe methods of follow-up  *Case-control study*—Give the eligibility criteria, and the sources and methods of case ascertainment and control selection. Give the rationale for the choice of cases and controls  *Cross-sectional study*—Give the eligibility criteria, and the sources and methods of selection of participants | Study Population |
|  |  | (*b*) *Cohort study*—For matched studies, give matching criteria and number of exposed and unexposed  *Case-control study*—For matched studies, give matching criteria and the number of controls per case | NA |
| Variables | 7 | Clearly define all outcomes, exposures, predictors, potential confounders, and effect modifiers. Give diagnostic criteria, if applicable | Examinations |
| Data sources/ measurement | 8 | For each variable of interest, give sources of data and details of methods of assessment (measurement). Describe comparability of assessment methods if there is more than one group | Examinations; RR intervals acquisition, processing and analysis of heart rate variability |
| Bias | 9 | Describe any efforts to address potential sources of bias | Results |
| Study size | 10 | Explain how the study size was arrived at | Study Population |
| Quantitative variables | 11 | Explain how quantitative variables were handled in the analyses. If applicable, describe which groupings were chosen and why | RR intervals acquisition, processing and analysis of heart rate variability; Machine learning algorithm and features importance |
| Statistical methods | 12 | (*a*) Describe all statistical methods, including those used to control for confounding | Machine learning algorithm and features importance |
|  |  | (*b*) Describe any methods used to examine subgroups and interactions | NA |
|  |  | (*c*) Explain how missing data were addressed | RR intervals acquisition, processing and analysis of heart rate variability |
|  |  | (*d*) *Cohort study*—If applicable, explain how loss to follow-up was addressed  *Case-control study*—If applicable, explain how matching of cases and controls was addressed  *Cross-sectional study*—If applicable, describe analytical methods taking account of sampling strategy | NA |
|  |  | (*e*) Describe any sensitivity analyses | Result; Discussion |
| **Results** | | | |
| Participants | 13* | (a) Report numbers of individuals at each stage of study—eg numbers potentially eligible, examined for eligibility, confirmed eligible, included in the study, completing follow-up, and analysed | Study Population; Results |
|  |  | (b) Give reasons for non-participation at each stage | Results |
|  |  | (c) Consider use of a flow diagram | NA |
| Descriptive data | 14* | (a) Give characteristics of study participants (eg demographic, clinical, social) and information on exposures and potential confounders | Results |
|  |  | (b) Indicate number of participants with missing data for each variable of interest | NA |
|  |  | (c) *Cohort study*—Summarise follow-up time (eg, average and total amount) | NA |
| Outcome data | 15* | *Cohort study*—Report numbers of outcome events or summary measures over time | Results |
|  |  | *Case-control study—*Report numbers in each exposure category, or summary measures of exposure | NA |
|  |  | *Cross-sectional study—*Report numbers of outcome events or summary measures | NA |
| Main results | 16 | (*a*) Give unadjusted estimates and, if applicable, confounder-adjusted estimates and their precision (eg, 95% confidence interval). Make clear which confounders were adjusted for and why they were included | Results |
|  |  | (*b*) Report category boundaries when continuous variables were categorized | NA |
|  |  | (*c*) If relevant, consider translating estimates of relative risk into absolute risk for a meaningful time period | NA |
| Other analyses | 17 | Report other analyses done—eg analyses of subgroups and interactions, and sensitivity analyses | Results |
| **Discussion** | | | |
| Key results | 18 | Summarise key results with reference to study objectives | Discussion; Conclusion |
| Limitations | 19 | Discuss limitations of the study, taking into account sources of potential bias or imprecision. Discuss both direction and magnitude of any potential bias | Discussion |
| Interpretation | 20 | Give a cautious overall interpretation of results considering objectives, limitations, multiplicity of analyses, results from similar studies, and other relevant evidence | Conclusion |
| Generalisability | 21 | Discuss the generalisability (external validity) of the study results | Discussion; Conclusion |
| **Other Information** | | | |
| Funding | 22 | Give the source of funding and the role of the funders for the present study and, if applicable, for the original study on which the present article is based | Funding |

TRIPOD+AI (Transparent Reporting of a Multivariable Prediction Model for Individual Prognosis or Diagnosis)

| **Section/topic** | **Item** | **Development/**  **evaluation*** | **Checklist item** | **Section** |
| --- | --- | --- | --- | --- |
| **Title** | | | | |
| Title | 1 | D;E | Identify the study as developing or evaluating the performance of a multivariable prediction model, the target population, and the outcome to be predicted | Title |
| **Abstract** | | | | |
| Abstract | 2 | D;E | See TRIPOD+AI for Abstracts checklist | NA |
| **Introduction** | | | | |
| Background | 3a | D;E | Explain the healthcare context (including whether diagnostic or prognostic) and rationale for developing or evaluating the prediction model, including references to existing models | Introduction |
|  | 3b | D;E | Describe the target population and the intended purpose of the prediction model in the context of the care pathway, including its intended users (eg, healthcare professionals, patients, public) | Introduction |
|  | 3c | D;E | Describe any known health inequalities between sociodemographic groups | NA |
| Objectives | 4 | D;E | Specify the study objectives, including whether the study describes the development or validation of a prediction model (or both) | Introduction |
| **Methods** | | | | |
| Data | 5a | D;E | Describe the sources of data separately for the development and evaluation datasets (eg, randomised trial, cohort, routine care or registry data), the rationale for using these data, and representativeness of the data | Study Population |
|  | 5b | D;E | Specify the dates of the collected participant data, including start and end of participant accrual; and, if applicable, end of follow-up | Examinations |
| Participants | 6a | D;E | Specify key elements of the study setting (eg, primary care, secondary care, general population) including the number and location of centres | Examinations |
|  | 6b | D;E | Describe the eligibility criteria for study participants | Study Population |
|  | 6c | D;E | Give details of any treatments received, and how they were handled during model development or evaluation, if relevant | NA |
| Data preparation | 7 | D;E | Describe any data pre-processing and quality checking, including whether this was similar across relevant sociodemographic groups | RR intervals acquisition, processing and analysis of heart rate variability |
| Outcome | 8a | D;E | Clearly define the outcome that is being predicted and the time horizon, including how and when assessed, the rationale for choosing this outcome, and whether the method of outcome assessment is consistent across sociodemographic groups | Examinations |
|  | 8b | D;E | If outcome assessment requires subjective interpretation, describe the qualifications and demographic characteristics of the outcome assessors | NA |
|  | 8c | D;E | Report any actions to blind assessment of the outcome to be predicted | RR intervals acquisition, processing and analysis of heart rate variability |
| Predictors | 9a | D | Describe the choice of initial predictors (eg, literature, previous models, all available predictors) and any pre-selection of predictors before model building | Examinations; RR intervals acquisition, processing and analysis of heart rate variability |
|  | 9b | D;E | Clearly define all predictors, including how and when they were measured (and any actions to blind assessment of predictors for the outcome and other predictors) | Examinations; RR intervals acquisition, processing and analysis of heart rate variability |
|  | 9c | D;E | If predictor measurement requires subjective interpretation, describe the qualifications and demographic characteristics of the predictor assessors | NA |
| Sample size | 10 | D;E | Explain how the study size was arrived at (separately for development and evaluation), and justify that the study size was sufficient to answer the research question. Include details of any sample size calculation | Study Population |
| Missing data | 11 | D;E | Describe how missing data were handled. Provide reasons for omitting any data | RR intervals acquisition, processing and analysis of heart rate variability |
| Analytical methods | 12a | D | Describe how the data were used (eg, for development and evaluation of model performance) in the analysis, including whether the data were partitioned, considering any sample size requirements | Machine learning algorithm and features importance |
|  | 12b | D | Depending on the type of model, describe how predictors were handled in the analyses (functional form, rescaling, transformation, or any standardisation) | Machine learning algorithm and features importance |
|  | 12c | D | Specify the type of model, rationale, all model building steps, including any hyperparameter tuning, and method for internal validation | Machine learning algorithm and features importance |
|  | 12d | D;E | Describe if and how any heterogeneity in estimates of model parameter values and model performance was handled and quantified across clusters (eg, hospitals, countries). See TRIPOD-Cluster for additional considerations‡ | NA |
|  | 12e | D;E | Specify all measures and plots used (and their rationale) to evaluate model performance (eg, discrimination, calibration, clinical utility) and, if relevant, to compare multiple models | Machine learning algorithm and features importance |
|  | 12f | E | Describe any model updating (eg, recalibration) arising from the model evaluation, either overall or for particular sociodemographic groups or settings | NA |
|  | 12g | E | For model evaluation, describe how the model predictions were calculated (eg, formula, code, object, application programming interface) | Machine learning algorithm and features importance |
| Class imbalance | 13 | D;E | If class imbalance methods were used, state why and how this was done, and any subsequent methods to recalibrate the model or the model predictions | NA |
| Fairness | 14 | D;E | Describe any approaches that were used to address model fairness and their rationale | NA |
| Model output | 15 | D | Specify the output of the prediction model (eg, probabilities, classification). Provide details and rationale for any classification and how the thresholds were identified | Introduction; Examinations |
| Training versus evaluation | 16 | D;E | Identify any differences between the development and evaluation data in healthcare setting, eligibility criteria, outcome, and predictors | Machine learning algorithm and features importance |
| Ethical approval | 17 | D;E | Name the institutional research board or ethics committee that approved the study and describe the participant informed consent or the ethics committee waiver of informed consent | Study Population |
| **Open science** | | | | |
| Funding | 18a | D;E | Give the source of funding and the role of the funders for the present study | Funding |
| Conflicts of interest | 18b | D;E | Declare any conflicts of interest and financial disclosures for all authors | Conflict of Interest |
| Protocol | 18c | D;E | Indicate where the study protocol can be accessed or state that a protocol was not prepared | NA |
| Registration | 18d | D;E | Provide registration information for the study, including register name and registration number, or state that the study was not registered | NA |
| Data sharing | 18e | D;E | Provide details of the availability of the study data | Data Availability Statement |
| Code sharing | 18f | D;E | Provide details of the availability of the analytical code§ | NA |
| **Patient and public involvement** | | | | |
| Patient and public involvement | 19 | D;E | Provide details of any patient and public involvement during the design, conduct, reporting, interpretation, or dissemination of the study or state no involvement | NA |
| **Result** | | | | |
| Participants | 20a | D;E | Describe the flow of participants through the study, including the number of participants with and without the outcome and, if applicable, a summary of the follow-up time. A diagram may be helpful | Results |
|  | 20b | D;E | Report the characteristics overall and, where applicable, for each data source or setting, including the key dates, key predictors (including demographics), treatments received, sample size, number of outcome events, follow-up time, and amount of missing data. A table may be helpful. Report any differences across key demographic groups | Results |
|  | 20c | E | For model evaluation, show a comparison with the development data of the distribution of important predictors (demographics, predictors, and outcome) | Results |
| Model development | 21 | D;E | Specify the number of participants and outcome events in each analysis (eg, for model development, hyperparameter tuning, model evaluation) | Results |
| Model specification | 22 | D | Provide details of the full prediction model (eg, formula, code, object, application programming interface) to allow predictions in new individuals and to enable third party evaluation and implementation, including any restrictions to access or reuse (eg, freely available, proprietary)¶ | Not reported |
| Model performance | 23a | D;E | Report model performance estimates with confidence intervals, including for any key subgroups (eg, sociodemographic). Consider plots to aid presentation | Not reported |
|  | 23b | D;E | If examined, report results of any heterogeneity in model performance across clusters. See TRIPOD-Cluster for additional details‡ | NA |
| Model updating | 24 | E | Report the results from any model updating, including the updated model and subsequent performance | NA |
| **Discussion** | | | | |
| Interpretation | 25 | D;E | Give an overall interpretation of the main results, including issues of fairness in the context of the objectives and previous studies | Discussion |
| Limitations | 26 | D;E | Discuss any limitations of the study (such as a non-representative sample, sample size, overfitting, missing data) and their effects on any biases, statistical uncertainty, and generalisability | Discussion |
| Usability of the model in the context of current care | 27a | D | Describe how poor quality or unavailable input data (eg, predictor values) should be assessed and handled when implementing the prediction model | NA |
|  | 27b | D | Specify whether users will be required to interact in the handling of the input data or use of the model, and what level of expertise is required of users | NA |
|  | 27c | D;E | Discuss any next steps for future research, with a specific view to applicability and generalisability of the model | Discussion; Conclusion |

# 2 Supplementary Data

Supplementary Table 1 provides a detailed list and interpretations of both the demographic and Heart Rate Variability (HRV) features used in the modeling process.

The HRV parameters were calculated using the neurokit2 Python package from data measured in both the supine and standing positions. Ten datasets were created by extracting parameters from RR interval (RRi) of 60, 120, 180, 240, and 300 seconds.

All datasets across the various time windows and measurement positions are derived from the comprehensive list of potential features described in Supplementary Table 1. The specific composition of each dataset including the final number of features used, which parameters were excluded, and the rationale for those exclusions is detailed in Supplementary Table 2. As there were no missing data for demographic variables, imputation procedures were not required.

# 3 Supplementary Tables

**Supplementary Table 1.** Parameters used in modeling process.

| Feature Name | Description |
| --- | --- |
| **DEMOGRAPHY DATA** | |
| Age | The chronological age of the player. |
| Height | The standing height of the player. |
| Weight | The total body mass of the player. |
| BMI | Body Mass Index |
| FATP | Body Fat Percentage, representing the proportion of total body weight that is fat tissue [%]. |
| FATM | Fat Mass, the absolute amount of fat tissue in the body [kg]. |
| PMM | Pure Muscle Mass [kg]. |
| BONEM | Bone Mass, representing the total weight of bone mineral in the body [kg]. |
| FFM | Fat-Free Mass, representing all body components except fat [kg]. |
| TBW | Total Body Water, the total amount of fluid contained within the body [kg]. |
| BMR | Basal Metabolic Rate, the amount of energy expended while at rest in a neutrally temperate environment [kJ/day]. |
| Maturity | The biological or physiological developmental stage of the subject |
| Experience_in_years | The total number of years the subject has been training at least three times a week. |
| Match_status (starter) | A binary indicator specifying whether the subject is a primary/starting player. |
| **HEART RATE VARIABILITY PARAMETERS** | |
| Time domain | |
| MeanNN | The mean of the RR intervals. |
| SDNN | The standard deviation of the RR intervals. |
| RMSSD | The square root of the mean of the squared successive differences between adjacent RR intervals. |
| CVNN | The standard deviation of the RR intervals (SDNN) divided by the mean of the RR intervals (MeanNN). |
| CVSD | The root mean square of successive differences (RMSSD) divided by the mean of the RR intervals (MeanNN). |
| SDRMSSD | SDNN/RMSSD, a time-domain equivalent for the low Frequency-to-High Frequency (LF/HF) Ratio. |
| pNN50 | The percentage of absolute differences in successive RRi greater than 50 ms. |
| Frequency domain | |
| LF | The spectral power of low frequencies (0.04 to 0.15 Hz). |
| HF | The spectral power of high frequencies (0.15 to 0.4 Hz). |
| VHF | The spectral power of very high frequencies (0.4 to 0.5 Hz). |
| TP | The total spectral power. |
| LFn | The normalized low frequency, obtained by dividing the low frequency power by the total power. |
| HFn | The normalized high frequency, obtained by dividing the high frequency power by the total power. Not conventional HF/(LF + HF) |
| LnHF | The log transformed HF. |
| Nonlinear | |
| SD1 | Standard deviation perpendicular to the line of identity. |
| SD2 | Standard deviation along the identity line. Index of long-term HRV changes. |
| SD1/SD2 | Ratio of SD1 to SD2. |
| GI | Guzik’s Index. |
| SI | Slope Index. |
| AI | Area Index. |
| PI | Porta’s Index. |
| SD1d | Short-term variance of contributions of decelerations (prolongations of RR intervals). |
| SD1a | Short-term variance of contributions of accelerations (shortenings of RR intervals). |
| C1d | The contributions of heart rate decelerations to short-term HRV. |
| C1a | The contributions of heart rate accelerations to short-term HRV. |
| SD2d | Long-term variance of contributions of decelerations (prolongations of RR intervals). |
| SD2a | Long-term variance of contributions of accelerations (shortenings of RR intervals). |
| C2d | The contributions of heart rate decelerations to long-term HRV. |
| C2a | The contributions of heart rate accelerations to long-term HRV. |
| SDNNd | Total variance of contributions of decelerations (prolongations of RR intervals). |
| SDNNa | Total variance of contributions of accelerations (shortenings of RR intervals). |
| Cd | The total contributions of heart rate decelerations to HRV. |
| Ca | The total contributions of heart rate accelerations to HRV. |
| PIP | Percentage of inflection points of the RR intervals series. |
| IALS | Inverse of the average length of the acceleration/deceleration segments. |
| PSS | Percentage of short segments. |
| PAS | Percentage of NN intervals in alternation segments. |
| DFA_alpha1 | The monofractal detrended fluctuation analysis of the HR signal, corresponding to short-term correlations. |
| DFA_alpha2 | The monofractal detrended fluctuation analysis of the HR signal, corresponding to long-term correlations. |
| ApEn | Approximate entropy. |
| SampEn | Sample entropy. |
| ShanEn | Shannon entropy. |
| FuzzyEn | Fuzzy entropy. |
| MSEn | Multiscale entropy. |
| CMSEn | Composite Multiscale entropy. |
| RCMSEn | Refined Composite Multiscale entropy. |
| CD | Correlation Dimension. |
| HFD | Higuchi’s Fractal Dimension. |
| KFD | Katz’s Fractal Dimension. |
| LZC | Lempel-Ziv Complexity. |
| SymDynMaxMin_0V | Percentage of words in the Max–min method that fall into the 0V family, representing sequences where all three consecutive symbols are equal. This method uses six levels of uniform quantization. |
| SymDynMaxMin_1V | Percentage of words in the Max–min method that fall into the 1V family, which includes sequences with only one variation among three consecutive symbols. |
| SymDynMaxMin_2LV | Percentage of words in the Max–min method that fall into the 2LV family, representing sequences with two variations in the same direction, forming an increasing or decreasing sequence. |
| SymDynMaxMin_2UV | Percentage of words in the Max–min method that fall into the 2UV family, where symbols vary two times in opposite directions, forming a peak or a valley. |
| SymDynSigma_0V | Percentage of words in the σ method that fall into the 0V family. The σ method uses three levels defined by the signal average and its variations shifted up and down by a set factor. |
| SymDynSigma_1V | Percentage of words in the σ method that fall into the 1V family. |
| SymDynSigma_2LV | Percentage of words in the σ method that fall into the 2LV family. |
| SymDynSigma_2UV | Percentage of words in the σ method that fall into the 2UV family. |
| SymDynEqualProba4_0V | Percentage of words using the Equal-probability method with four quantization levels (q = 4) that fall into the 0V family. |
| SymDynEqualProba4_1V | Percentage of words using the Equal-probability method with four quantization levels that fall into the 1V family. |
| SymDynEqualProba4_2LV | Percentage of words using the Equal-probability method with four quantization levels that fall into the 2LV family. |
| SymDynEqualProba4_2UV | Percentage of words using the Equal-probability method with four quantization levels that fall into the 2UV family. |
| SymDynEqualProba6_0V | Percentage of words using the Equal-probability method with six quantization levels (q = 6) that fall into the 0V family. |
| SymDynEqualProba6_1V | Percentage of words using the Equal-probability method with six quantization levels that fall into the 1V family. |
| SymDynEqualProba6_2LV | Percentage of words using the Equal-probability method with six quantization levels that fall into the 2LV family. |
| SymDynEqualProba6_2UV | Percentage of words using the Equal-probability method with six quantization levels that fall into the 2UV family. |

**Supplementary Table 2.** Details of used datasets

| **Dataset** | **Features** | **Excluded Features** | **Exclusion Reason** |
| --- | --- | --- | --- |
| Baseline model | 14 | Heart Rate Variability Parameters | Only demography data |
| Supine 60s | 77 | DFA_alpha2, MSEn, SampEn | Neurokit2 package returned infinite (inf) values during calculation |
| Standing 60s | 79 | DFA_alpha2 | Neurokit2 package returned infinite (inf) values during calculation |
| Supine 120s | 79 | DFA_alpha2 | Neurokit2 package returned infinite (inf) values during calculation |
| Standing 120s | 79 | DFA_alpha2 | Neurokit2 package returned infinite (inf) values during calculation |
| Supine 180s | 80 | NA | NA |
| Standing 180s | 80 | NA | NA |
| Supine 240s | 80 | NA | NA |
| Standing 240s | 80 | NA | NA |
| Supine 300s | 80 | NA | NA |
| Standing 300s | 80 | NA | NA |
